# Comparison of safety, efficacy, and cost effectiveness of benzyl benzoate, permethrin, and ivermectin in patients of scabies

**DOI:** 10.4103/0253-7613.48882

**Published:** 2009-02

**Authors:** Narendra P. Bachewar, Vijay R. Thawani, Smita N. Mali, Kunda J. Gharpure, Vaishali P. Shingade, Ganesh N. Dakhale

**Affiliations:** JMF's ACPM Med. College, Dhule, India; 1 Government Medical College, Nagpur, India; 2 Indira Gandhi Government Medical College, Nagpur, India

**Keywords:** Efficacy, economic analysis, scabies, treatment

## Abstract

**Objective:**

To compare three treatment modalities in scabies for safety, efficacy, and economy in a local population of Nagpur.

**Materials and Methods:**

This was a prospective, randomized, comparative clinical trial conducted in 103 participants, randomly allocated to three groups. First group received benzyl benzoate (BB) 25% lotion, second group received permethrin 5% cream, whereas third group received tablet ivermectin 200 μg/kg as a single dose. The participants were recalled after one week for follow-up evaluation. If there were no signs of cure, the same intervention was repeated. The participants were followed up for two weeks for cure rate, adverse drug reaction (ADR) monitoring, and postintervention observation. The follow-up was stopped after two weeks.

**Statistics:**

Fischer's exact test using Graph pad Instat v 3.05.

**Results:**

Ivermectin showed 100% cure rate after two weeks of treatment. Permethrin decreased pruritus by 76% at the end of one week and had significantly better cure rate than ivermectin. At the end of two weeks treatment, this finding was reversed, that is, cure rate in ivermectin group was 100%. For cost-effectiveness analysis, treatment regimens were formulated hypothetically for comparison from Markov population tree for decision analysis. It was found that BB and ivermectin each consecutively for two weeks were most cost effective regimens giving complete cure in four weeks, while ivermectin was the fastest regimen giving the same results in two weeks.

**Conclusion:**

Benzyl benzoate as first line intervention and ivermectin in the remaining gave best cost-effective results in the study patients of scabies.

## Introduction

The annual incidence of scabies being three million, the disease burden on the developing countries justifies this study[1,2] in India. Scabies is of greater importance due to its affinity for economically marginalized and immunocompromised hosts.[3] *Sarcoptes scabie* gains its importance by making the host morbid by its lesions, immense pruritus, high infectivity, frequent relapses, persistence of symptoms for many days even after eradication, and resistance to the routine treatment.[4] Treatment of scabies has changed from Celsus to modern medicine, from sulfur to permethrin and ivermectin.[5] Current treatment of scabies comprises of topical antiscabetics, applied all over the body for a specified contact period.[1] Research has been attempted to find the best antiscabetic focusing on efficacy and safety data.[6] Indian population being commonly affected by scabies, masses have poor affordability for the most efficacious antiscabetic. Hence, it was decided to study and compare three antiscabetics - the most commonly used benzyl benzoate, the currently considered medicine of choice permethrin, and the most recently introduced ivermectin – in the local population of Nagpur.

### Aim

To provide better and improved treatment option for scabies based on cost effectiveness, efficacy, and safety profile.

### Objectives

- To study the different available treatment modalities in scabies.
- To compare efficacy and cost effectiveness of three treatment modalities and suggest the best intervention for cure and to find most economical therapy.

## Materials and Methods

### Study design

This was a prospective, randomized, parallel, noncrossover, comparative, controlled clinical trial, approved by Ethics Committee of Government Medical College and Hospital, Nagpur (GMCN), conducted in patients attending the OPD of Skin and Venereal Disease Department of GMCN from 15^th^ March to 23^rd^ July, 2007. Dermatologist diagnosed patients of scabies were approached with request to participate in the trial with a detailed information sheet. Diagnosis of the disease was based on clinical symptoms and clinical history.

Patients willing to participate were screened by applying the inclusion and exclusion criteria. Inclusion criteria included newly diagnosed patients of scabies, of either gender, above 12 years of age, willing to participate, and give written informed consent. For inclusion, the patients had to satisfy at least three out of the five criteria viz. history of contact with a scabies patient, complaint of nocturnal itching, history of involvement of family members, presence of classical burrows on clinical examination, and presence of typical scabetic lesions like papules, nodules, or vesicles.

Exclusion criteria included pregnant or lactating women; women of child bearing age or planning for conception in near future; participants with abnormal liver and kidney functions, known thyroid disease, cardiac disorders, nervous system disorders, and psychiatric illnesses; and participants with history of diabetes mellitus, hypertension, or chronic infectious diseases. Participants taking any concurrent medication for other illness, consuming tobacco in any form, alcohol, or any substance of abuse were excluded from the trial. Participants with any other associated skin disease, which could alter the picture of scabies; known/suspected immunocompromised individuals, having scabies with atypical presentations like crusted scabies or scabies incognito; participants who had taken any antiscabetic treatment in the preceding week; and noncompliant participants were excluded. In this trial ivermectin was given as supervised[7] medication along with printed handouts of “do's” in the local vernacular language. During their second visit, those participants who failed to answer correctly the method of application followed by them and the treatment of fomite were considered as noncompliant.

Participants were first treated for the secondary infection, if present, with azithromycin 500 mg once daily for three days/ampicillin 500 mg 6 hourly for five days, as per clinical judgment of the senior dermatologist and subsequently included in the trial. The demographic data of the participants like age, gender, occupation, education, and marital status were collected in the case record form (CRF) by the principle investigator (PI). At each visit the participant was examined by two doctors, a dermatologist and the PI. The 103 enrolled participants were allocated to three groups according to random allocation number generated through computer and provided with any one of the chosen three therapeutic interventions. Participants were advised not to use or mix any other treatment, including antipruritic or antihistaminic medicines. All participants were issued 25% benzyl benzoate (BB) lotion for topical application for the family members and close contacts, as per the standard treatment guidelines to check the reinfestation of the trial participants. Participants were reinforced with the message about treatment of the family members and other close contacts during each visit.

### Interventions

- Benzyl benzoate 25% lotion to be applied and left overnight to the whole body below neck for two consecutive nights. The BB 50 ml lotion sealed and capped amber colored glass bottle (M/s Deepti Pharmaceuticals, MIDC, Nagpur), was supplied by Government of Maharashtra. In local market it was priced at Indian rupee (INR) 11.
- Permethrin 5% cream to be applied once to the whole body below neck and left overnight. The commercially available permethrin Permite™ BP: 30 g cream, carton covered, capped aluminum tube (M/s Encube Ethical Private Limited, Madkaim Industrial estate, Ponda, Goa), was priced at INR60. Each tube contained 1500 mg active permethrin, 30 mg formaldehyde solution, and cream base.
- Tablet ivermectin 200 *μ*g/kg to be consumed as a single dose. The commercially available ivermectin Vermectin™ BP: 6 mg/12 mg scored uncoated tablets, supplied in carton covered blister pack of single tablet (M/s Micro Labs Limited, SIPCOT Industrial Complex, Hosur, Tamil Nadu), was priced at INR25. Each tablet contained yellow oxide of iron as coloring agent.

Participants on intervention I and II were advised to bathe with warm water before application of medication and on subsequent morning. The participants were recalled after one week for follow-up evaluation. The same intervention was repeated if there were no signs of improvement. The participants were followed up for two weeks for cure rate, adverse drug reaction (ADR) monitoring, and postintervention observation. Because of the known safety of all the three interventions, no laboratory examinations were advised. The follow-up was stopped after two weeks subject to achievement of one of the three endpoints – participant defined as completely cured, participant who developed severe ADR, or participant who was not cured two weeks postintervention follow up.

### Cure

During the one week postintervention follow up, both the doctors examined each participant. The participants who did not have any new lesions were considered as cured. Papules, vesicles, and classical burrows were considered as new lesions suggestive of live parasite. The cured participants were prescribed antihistaminic for symptomatic treatment of remaining pruritus and the uncured participants were prescribed repeat intervention along with antihistaminic. All the cured as well as uncured participants were again called after one week for the follow-up examination and recording of the parameters.

### Pruritus

All participants were followed up for one week postintervention, for improvement of pruritus. Antipruritic medicines, if needed, were prescribed only after this. After one week follow up, each participant was asked to quote 0/25/50/75/100%, on the visual analogue scale (VAS) about the percent of remaining pruritus, considering the pruritus at first visit as 100%. Thus, pruritus was observed for only one week postintervention. On each visit, the participants were asked for occurrence of any ADR.

### Statistical analysis

Fisher's exact test was applied using Graph pad Instat version 3.05. The ‘*P*’ value of less than 0.05 was considered as statistically significant. The sample size was calculated taking into consideration the future dropouts and was based on previous similar studies.[6]

## Results

While doing randomized sampling for this study, gender-wise distribution and the follow-up in all the three treatment groups was maintained throughout the trial, except the BB group in which we experienced lower follow up in women. The same ratios as in followed-up groups were maintained in cure rates at the end of two weeks [Table 1].

**Table 1:** Comparison of gender ratios at various stages of clinical trial in the treatment groups

	*Benzyl benzoate (M/F = Ratio*)	*Permethrin (M/F = Ratio)*	*Ivermectin (M/F = Ratio)*	*Total (M/F = Ratio)*
Participated	23/12 = 1.917	22/12 = 1.833	18/16 = 1.125	63/40 = 1.575
Followed up	18/7 = 2.285	18/10 = 1.8	14/13 = 1.077	50/30 = 1.667
Cured	16/7 = 2.570	17/10 = 1.7	14/13 = 1.077	47/30 = 1.567

Living status – living singly or with a partner – did not show any implication over cure rates with any of the treatments. Majority (84%) of the participants were from 12–41 years age group, showing the usual trend of scabies.[6] Most (85%) of the participants had at least primary education. This confirms their literacy and ability to understand the written instructions provided to them. The distribution of typical scabies lesions over different body parts was variable. To enumerate the important ones, pubic region lead with 18%, followed by abdomen, wrists, and web space affliction amounting 13% each, and cubital region was affected in 11%. Ivermectin was least promising at the end of one-week treatment, but it showed 100% cure rate after two-week treatment [Table 2].

**Table 2:** Participant response during the trial in each group

*Participant response*	*BB*	*P*	*I*
Recruited participants	35	34	34
Followed up at the end of two weeks	25	28	27
Cure rate at end of one week (%)	76	82.14	55.56
Cure rate at the end of two weeks (%)	92	96.43	100

BB - benzyl benzoate, P - permethrin, I - ivermectin

Considering pruritus as presenting symptom, the medication which alleviates it has greater acceptance in clinical practice. We observed that permethrin qualifies for this, with 76% decrease in pruritus at the end of one week. Statistical analysis with Fisher's exact test showed that at the end of one week, considering the cure rate, only permethrin was significantly (*P* < 0.05) better than ivermectin. At the end of two-week treatment, this finding was reversed because the cure rate in ivermectin group was 100%.

### Economic analysis

For this we compared the three interventions on economic basis and cost effectiveness. The following methods were used.

### Cost effective analysis

On the basis of total expenditure incurred on medicines at the end of two weeks in INR and cure rate in percentage, the cost effectiveness was calculated and the three interventions were compared on the basis of amount needed to treat one case successfully.

### Cost-effectiveness graph

**Figure 1:**
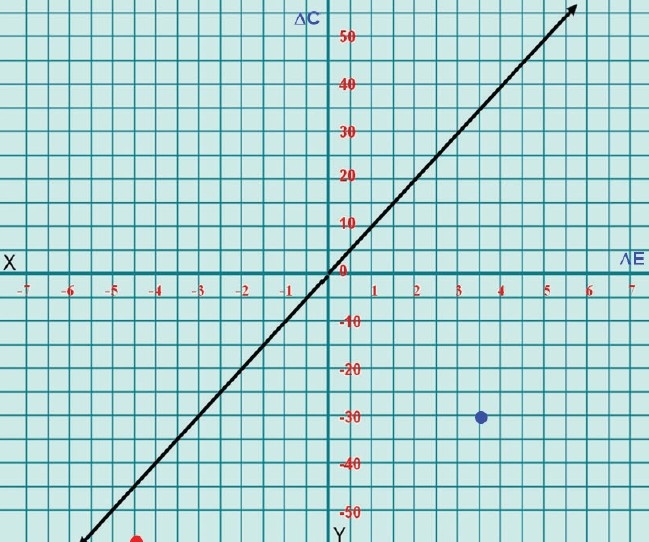
Cost-effectiveness of benzyl benzoate and ivermectin as compared to permethrin ΔC - incremental cost, ΔE - incremental efficacy, Red dot - cost effectiveness of ivermectin (ΔC = − INR57.08; ΔE = −4.43), Blue dot - cost effectiveness of benzyl benzoate (ΔC = −INR34.61; ΔE = 3.57)

In this graph [Figure 1] x-axis shows ΔE, that is, incremental effectiveness, y-axis shows ΔC, that is, incremental cost, and λ-line is the arbitrary line differentiating between the zones (zone above the λ-line is unacceptability zone and zone below the line is acceptability zone).

Incremental cost = cost of the new (second) treatment – cost of the old (first) treatment

Incremental effectiveness = efficacy of new (second) treatment – efficacy of old (first) treatment

Considering the cure rates at the end of one week, cost incurred to treat 100 participants for different interventions was found to be:

Total cost of treatment = INR for participants cured with one week treatment + INR for participants who were treated for two weeks

Cost of one bottle of BB was INR11, for one permethrin tube was INR60, and that of one 12 mg tablet of ivermectin was INR25. With these costs the cost of treatment in INR was calculated as:

Benzyl benzoate = (11 × 76) + (22 × 24) = 1364

Permethrin = (60 × 82.14) + (120 × 17.86) = 7071.6

Ivermectin = (25 × 55.56) + (50 × 44.44) = 3611

Amount needed to treat one case of scabies successfully at the end of one week was least for BB with INR14.47 and it was most for permethrin with INR73.04 [Table 3].

**Table 3:** Cost-effectiveness analysis of the trial medicines at the end of one week

*Parameters*	*BB*	*P*	*I*
Cost in INR for 100 participants	11 × 100 = 1100	60 × 100 = 6000	25 × 100 = 2500
Cure rate (%) (effectiveness)	76	82.14	55.56
Cost effectiveness	INR1100 for 76 participants	INR6000 for 82.14 participants	INR2500 for 55.56 participants
Cost to treat one case (INR)	14.47	73.04	45

BB - benzyl benzoate, P - permethrin, I - ivermectin

All the values thus obtained were used for further analysis. The same parameter at the end of two weeks was again least for BB with INR14.83 and was most for permethrin with INR73.33. Thus, permethrin was approximately five times costlier for treating one scabies case successfully at the end of one- or two-week treatment [Table 4]

**Table 4:** Cost-effectiveness analysis of the trial medicines at the end of two weeks

*Parameters*	*BB*	*P*	*I*
Cost in INR for 100 participants	1364	7071.6	3611
Cure rate (%) (effectiveness)	92	96.43	100
Cost effectiveness	INR1364 for 92 participants	INR7071.6 for 96.43 participants	INR3611 for 100 participants
Cost to treat one case (INR)	14.83	73.33	36.11

BB - benzyl benzoate, P - permethrin, I - ivermectin

The cost-effectiveness graphs confirm that as compared to permethrin, BB and ivermectin fall in acceptability zone. Ivermectin falls at the center of acceptability zone, thereby suggesting that it is more cost effective than BB [Figure 1].

### Markov model of decision analysis[8,9]

**Figure 2:**
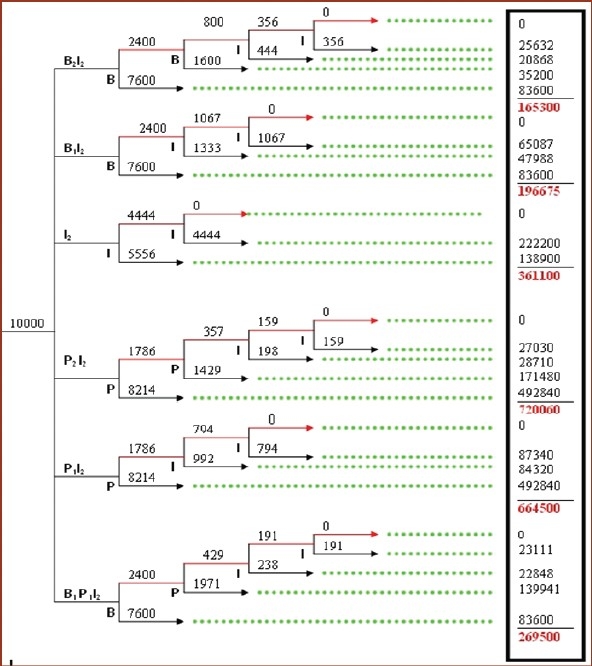
Markov model of decision analysis for cost effectivness of different regimens in scabies benzyl benzoate, P - permethrin, I - ivermectin treatment, Red line - not cured, Black line - cured, Green dotted line - tracing lines The “Markov population model of decision analysis” was prepared using our clinical data for efficacy of each intervention at one and two weeks. Figures in the box on the right side show cost of treatment. The numbers in subscript denote duration of treatment in weeks. The tree has been designed considering 10,000 patient recruitments in each branch for easy analysis. Numbers above the horizontal arrow lines show number of patients in that branch.

“Markov model of decision analysis” is a designed tree in which detailed cost at each step is given at the end of the tree and total cost of each limb is calculated. Each step follows the cure rate of that medicine at that step. We calculated the cost of treatment according to efficacy of each medicine at the end of one and two weeks as per the results obtained [Figure 2].

In this trial the cure rate of ivermectin was found to be 100% at the end of two-week treatment. We formulated various regimens after permutations and combinations to gain 100% cure rate for the treatment of scabies patients in the tertiary care institution level. We formulated the regimens considering the cure rates of all the three medicines of this trial, which we got at the end of one- and two-week treatment. The hypothetical regimens were:

1. B_2_I_2_
2. B_1_I_2_
3. I_2_
4. P_2_I_2_
5. P_1_I_2_
6. B_1_P_1_I_2_

where subscript denotes the number of weeks for which the treatment is given. The alphabets denote the medicine given for that duration, for example, B_2_I_2_ means benzyl benzoate treatment given for two weeks and then uncured patients treated with ivermectin treatment for next two weeks.

Among all the regimens, ivermectin had the shortest duration of two weeks. All the regimens have two-week treatment with ivermectin at the end because in this trial we found ivermectin having 100% cure rate at the end of two weeks. None of the other two medicines offered such a cure rate.

Markov population model of decision analysis shows that B_2_I_2_ is the cheapest regimen, while I_2_ gives the fastest results in half the duration with double the cost of B_2_I_2_. The third one which falls in between is, B_1_I_2_, also a cost-effective regimen giving 100% cure in three weeks.

## Discussion

### Cure rates

The 100% cure rate was obtained with oral ivermectin at the end of two weeks. Some of the previous studies have reported similar results,[6,10–12] but the interesting finding was the lesser decrease in pruritus score with ivermectin as compared to permethrin. None of the previous studies have reported the superior efficacy of ivermectin.[12,13] It is presumed that the VAS used for recording of residual pruritus may have suffered due to the subjective variation, but then VAS was the best scale that could have been used in this study.[14,15] Cure rates of participants in BB group and permethrin group were in the same range as concluded by previous researchers.[6,11,12,16,17]

### Economic studies

The limiting factor in the wide use of permethrin is its cost.[5,18,11] In another study on cost benefit and safety analysis of antiscabetic drugs, 5% permethrin was claimed to be the best option for scabies in infants and young children.[19] But that study was influenced by cost as well as safety. In addition, the study was done in infants; hence, we cannot compare our data with it. Concerns have been raised about the use of ivermectin in young children and pregnant women, because there may be more drug penetration through the immature blood-brain barrier.[20]

### Safety

In our study, neither patients nor investigators noted any ADR, alike other studies.[11,13,19,20] The review of literature reveals significant statistical association between use of ivermectin and higher mortality and recommends that ivermectin should not be used for treatment of scabies in the elderly, pending confirmation of association being causal or serendipitous.[21] However, many researchers have criticized the methodology and the conclusion of that study and a FDA review of the study found no causal relationship between the deaths and ivermectin.[21,22]

### Finding the best option

Karthikeyan *et al*, gave definition for an ideal antiscabetic, as that, which is effective against adult and egg, easily applicable, nonsensitizing, nonirritating, nontoxic, and economical.[5] Johnston *et al*, opined that the treatment selected should be determined by local epidemiology of resistance, drug toxicity, and particularly in underdeveloped countries, cost and availability.[23] Chosidow *et al*, have recommended that ivermectin should be routinely used in patients who do not show response to a topical antiscabetic and it may be the appropriate first choice for the elderly, patients with generalized eczema, and others who may be unable to tolerate or comply with topical agents.[4] Chosidow *et al*, have admitted that optimal therapy for classic scabies is not certain. Topical treatment may be poorly tolerated. Oral ivermectin is convenient, but the optimal dosing regimen remains uncertain.[4] A fact sheet for patients that is available from the Center for Drug Control, USA, recommends the use of 5% permethrin as standard therapy with crotamiton or oral ivermectin as alternatives.[4]

Ivermectin is the future drug of choice for scabies.[5] In France ivermectin is licensed for human scabies since 2001.[24] The treatment is easy, quick, safe, and well tolerated with maximal patient compliance.[24] Our findings show that BB required the least INR to treat one case of scabies, ivermectin was the most cost effective single medicine by virtue of its 100% efficacy, and permethrin gave the fastest symptomatic relief.

After these confounding findings, we tried various permutations and combinations of regimes to obtain optimum desirable results, which added to the novelty of this research. Safetywise, we found the three medicines equal as no ADR was reported with any of these. Thus, only the cost effectiveness played the decisive role in selection. On Markov tree for decision analysis,[8,9] we found BB as the preferred medicine. Our finding of B_2_I_2_ as the most economical regimen recommends that one should start with BB treatment for scabies afflicted for first two weeks and then shift the uncured patients to oral ivermectin therapy for next two weeks.

In another study where oral ivermectin was comapred to topical 10% BB, it was found that absolute results favored the use of ivermectin, but the difference was not statistically significant.[20] A recent trial proved that ivermectin significantly decreased post-treatment reinfestation as compared to permethrin, but the cause remained obscure.[25] On the basis of such results many researchers, regulatory bodies, and WHO recommend ivermectin use for mass treatment of scabies.[26] Ivermectin cannot be the best drug in every hospital setting; in spite of showing promise in its efficacy and safety, as the local resistance pattern (which we did not find) and the affordability of patient population varies, and other cheaper alternatives still have an important role in marginalized economies. We agree that addition of ivermectin to the clinician's armamentarium should be welcomed for the treatment of scabies, as it is the most cost effective, safe medicine which gives relief in the shortest possible time.[22]

Currently, permethrin is commonly recommended as the drug of choice. But in our study it did not get that coveted position. If we evaluate the best symptomatic treatment which can give moderate cure rate, permethrin is the answer. It is also equally safe. However, we did not find it in our recommendation because of the usual practice of giving additional antipruritic along with antiscabetics right at the very first visit for symptomatic benefit to all patients. Additionally, the result of highest cure rate with permethrin, which we found at the end of one week, did not sustain after two weeks.

### Limitations

We admit that our study was biased due to nonblinding. Blinding was not possible because the formulations were different, that is, lotion, cream, and tablet. This study does have the inherent limitations in Markov population tree of decision analysis, like nonconsideration of lateral transfer of disease, reinfestation, and loss to the trial for meeting of any of the unusual endpoint.

## Conclusion

In Government hospital like ours, we recommend that each patient of scabies should be first advised BB topically for two weeks. Nonresponders should then be advised oral ivermectin once a week for two weeks. In case of affording patients ivermectin once a week for two weeks currently offers quick, better, and safe results, restricting further morbidity and secondary transmission. The novel research design of clinical trial with economic analysis is easy and accurate method for doing a pragmatic trial, which can be replicated in other diseases.
